# Prognostic and mechanistic potential of progesterone sulfates in intrahepatic cholestasis of pregnancy and pruritus gravidarum

**DOI:** 10.1002/hep.28265

**Published:** 2015-12-28

**Authors:** Shadi Abu‐Hayyeh, Caroline Ovadia, TinaMarie Lieu, Dane D. Jensen, Jenny Chambers, Peter H. Dixon, Anita Lövgren‐Sandblom, Ruth Bolier, Dagmar Tolenaars, Andreas E. Kremer, Argyro Syngelaki, Muna Noori, David Williams, Jose J.G. Marin, Maria J. Monte, Kypros H. Nicolaides, Ulrich Beuers, Ronald Oude‐Elferink, Paul T. Seed, Lucy Chappell, Hanns‐Ulrich Marschall, Nigel W. Bunnett, Catherine Williamson

**Affiliations:** ^1^ Women's Health Academic CentreKing's College LondonLondonUnited Kingdom; ^2^ Monash Institute of Pharmaceutical Sciences and Australian Research Council Centre of Excellence in Convergent Bio‐Nano Science and TechnologyMonash UniversityParkvilleVictoriaAustralia; ^3^ Institute of Reproductive and Developmental BiologyImperial College LondonLondonUnited Kingdom; ^4^ Department of Clinical ChemistryKarolinska University Hospital HuddingeStockholmSweden; ^5^ Tytgat Institute for Liver and Intestinal ResearchAcademic Medical CentreAmsterdamThe Netherlands; ^6^ Department of Medicine 1Friedrich‐Alexander‐University of Erlangen‐NurembergErlangenGermany; ^7^ Harris Birthright Research Centre for Fetal MedicineKing's College HospitalLondonUnited Kingdom; ^8^ Institute for Women's HealthUniversity College London HospitalsLondonUnited Kingdom; ^9^ Laboratory of Experimental Hepatology and Drug Targeting (HEVEFARM), Biomedical Research Institute of Salamanca (IBSAL)University of Salamanca, National Institute for the Study of Liver and Gastrointestinal Diseases (CIBERehd)SalamancaSpain; ^10^ Institute of Medicine, Department of Molecular and Clinical MedicineUniversity of GothenburgGothenburgSweden; ^11^ Department of PharmacologyUniversity of MelbourneParkvilleVictoriaAustralia

## Abstract

A challenge in obstetrics is to distinguish pathological symptoms from those associated with normal changes of pregnancy, typified by the need to differentiate whether gestational pruritus of the skin is an early symptom of intrahepatic cholestasis of pregnancy (ICP) or due to benign pruritus gravidarum. ICP is characterized by raised serum bile acids and complicated by spontaneous preterm labor and stillbirth. A biomarker for ICP would be invaluable for early diagnosis and treatment and to enable its differentiation from other maternal diseases. Three progesterone sulfate compounds, whose concentrations have not previously been studied, were newly synthesized and assayed in the serum of three groups of ICP patients and found to be significantly higher in ICP at 9‐15 weeks of gestation and prior to symptom onset (group 1 cases/samples: ICP n = 35/80, uncomplicated pregnancy = 29/100), demonstrating that all three progesterone sulfates are prognostic for ICP. Concentrations of progesterone sulfates were associated with itch severity and, in combination with autotaxin, distinguished pregnant women with itch that would subsequently develop ICP from pruritus gravidarum (group 2: ICP n = 41, pruritus gravidarum n = 14). In a third group of first‐trimester samples all progesterone sulfates were significantly elevated in serum from low‐risk asymptomatic women who subsequently developed ICP (ICP/uncomplicated pregnancy n = 54/51). Finally, we show mechanistically that progesterone sulfates mediate itch by evoking a Tgr5‐dependent scratch response in mice. *Conclusion:* Our discovery that sulfated progesterone metabolites are a prognostic indicator for ICP will help predict onset of ICP and distinguish it from benign pruritus gravidarum, enabling targeted obstetric care to a high‐risk population. Delineation of a progesterone sulfate‐TGR5 pruritus axis identifies a therapeutic target for itch management in ICP. (Hepatology 2016;63:1287–1298)

**Abbreviations**
cAMP: cyclic adenosine monophosphate
CAMYEL: cAMP sensor using YFP‐Epac‐RLuc
cDNA: complementary DNA
CI: confidence interval
FXR: farnesoid X receptor
ICP: intrahepatic cholestasis of pregnancy
KO: knockout
MeOH: methanol
OR: odds ratio
PG: pruritus gravidarum
PM2DiS: 5α‐pregnan‐3α,‐20α‐diol‐3,20‐disulfate
PM3DiS: 5β‐pregnan‐3α,‐20α‐diol‐3,20‐disulfate
PM3S: 5β‐pregnan‐3α,‐20α‐diol‐3‐sulfate
UDCA: ursodeoxycholic acid
WT: wild type

A major challenge for obstetricians is to distinguish serious disorders associated with increased maternal and fetal mortality from low‐risk gestational changes. Currently, the presenting symptoms of many obstetric syndromes are nonspecific with few early biomarkers of serious maternal disease. We aimed to address this problem for intrahepatic cholestasis of pregnancy (ICP), the commonest liver‐specific disorder of pregnancy.^1^ ICP is complicated by spontaneous preterm labor, fetal distress, and intrauterine death.^1^, ^2^ Early recognition of ICP is important to enable prompt treatment and appropriate pregnancy surveillance. The presenting symptom of ICP is pruritus (skin), and diagnosis is confirmed by demonstration of raised total serum bile acids. However, maternal pruritus without hepatic impairment or dermatological disorder (i.e., pruritus gravidarum [PG]) affects up to 25% of pregnant women,^3^, ^4^ while ICP is much less common. It has a variable geographic prevalence: In the United Kingdom ICP affects 0.7% of pregnant women but is twice as common in women of Indian or Pakistani origin,^5^ while in Chile it affects up to 4% of pregnant women.^6^

The etiology of gestational pruritus (both benign and in ICP) is not established. Several endogenous compounds have been proposed as biochemical mediators of pruritus in ICP, including lysophosphatidic acid, a neuronal activator that can act as a pruritogen, the formation of which is catalyzed by the enzyme autotaxin.^7^, ^8^ These molecules are raised in the serum of women with ICP after disease onset.^8^ The secondary bile acids deoxycholic acid and lithocholic acid can activate the G protein‐coupled receptor TGR5 on sensory nerves to stimulate release of itch‐selective neuropeptides in the spinal cord and evoke a Tgr5‐dependent itch response in mice.^9^ These results indicate that bile acids may induce pruritus but require further evaluation in ICP as secondary bile acids are not typically raised in the condition and concentrations of total maternal serum bile acids do not correlate with pruritus severity.^10^ Although studies of urine samples from ICP cases implicate progesterone sulfates as pruritogens,^11^ the precise structures of the compounds and their capacity to cause pruritus remain to be determined.

Sulfated progesterone metabolites contribute to the etiology of ICP; they are partial agonists of the bile acid receptor farnesoid X receptor (FXR)^12^ and competitively inhibit hepatic bile acid uptake^13^ and efflux,^14^ resulting in cholestasis and hypercholanemia.^12^ Serum concentrations of progesterone sulfates are elevated in women with ICP at 35‐41 weeks of gestation,^12^, ^15^ typically after diagnosis. We hypothesized that progesterone sulfates are raised in early pregnancy prior to the onset of ICP and thus are potential early biomarkers that can distinguish ICP from benign PG. We also hypothesized that they signal through TGR5 to mediate pruritus.

This study used three groups of ICP cases and pregnant controls to establish whether progesterone sulfates are biomarker candidates for ICP diagnosis prior to biochemical derangement and to evaluate their role and potential mechanism of action as pruritogens using *in vitro* and *in vivo* approaches. Our results reveal a key role for the progesterone sulfate‐TGR5 axis in ICP.

## Materials and Methods

#### Study Approval

This study conformed to the 1975 Declaration of Helsinki guidelines; permission was obtained from the ethics committees of Hammersmith Hospitals NHS Trust, London (97/5197 and 08/H0707/21), and King's College Hospitals NHS Trust, London (03WH06). Written informed consent was received from participants prior to inclusion in the study. Murine studies were approved by the Monash University Animal Ethics Committee.

#### Human Serum Samples

Serial blood samples were collected from three prospectively recruited groups of women with ICP, PG, or controls with uncomplicated pregnancies at intervals dependent upon gestation and patient attendance. Sample preparation was as described.^16^ Three separate patient groups were used, to ensure that results could be replicated.

Group 1 comprised 64 women: 35 opportunistically recruited “high‐risk” ICP cases with a history of cholestasis in a previous pregnancy and 29 with uncomplicated pregnancies. Women with ICP commenced ursodeoxycholic acid (UDCA) treatment per personal and practitioner preference following diagnosis (seven were untreated, six were treated with UDCA, and 22 were recruited untreated and subsequently UDCA‐treated). Group 2 (the pruritus group) comprised 55 women with skin pruritus in pregnancy, 41 of whom had pregnancies complicated by ICP (23 with previous ICP) and 14 of whom had normal pregnancies (nine with previous ICP); of the women who subsequently developed ICP, 14 provided serum samples prior to the onset of hypercholanemia (raised bile acids) but after the onset of pruritus. Women in groups 1 and 2 were recruited while undergoing antenatal care at the tertiary hospitals of Imperial College London or through the ICP Support charity. Cases were recruited between 2007 and 2014 and selected to include all cases where longitudinal samples were available; 20% of ICP cases were tertiary referrals (of this group 71% were referred from specialists in different UK regions and 29% were referred from the UK charity ICP Support).

To evaluate whether progesterone sulfate concentrations reduce after delivery, we identified postnatal serum samples from a subgroup of 12 ICP cases (due to the limited number of postnatal samples collected) and compared the concentration of progesterone sulfates with the third‐trimester serum sample.

Group 3 comprised 105 asymptomatic women at 11‐14 weeks' gestation, 54 of whom later developed ICP and 51 of whom subsequently had normal pregnancies. Women were recruited at aneuploidy screening at King's College Hospital, serum samples were taken, and clinical follow‐up by a research midwife identified women who developed ICP; the next sample taken from a woman with a normal pregnancy was then used as a control (serum analyses were incomplete due to technical error resulting in exclusion of three women with normal pregnancies).

All cases of ICP were confirmed by demonstration of serum bile acids ≥10 μmol/L, and some cases also had raised liver transaminases in association with pruritus and no additional identifiable cause for their liver dysfunction. Exclusion criteria were other causes of hepatic dysfunction, including preeclampsia; hemolysis, elevated liver enzymes, and low platelets (HELLP) syndrome; acute fatty liver of pregnancy; primary biliary cirrhosis; active viral hepatitis; any ultrasound abnormality that may result in biliary obstruction; and multifetal pregnancy.

#### Biophysical Profiling of Participants

Details of the participants' relevant previous medical history, family history, ethnicity, results of investigations, pregnancy, and delivery were taken throughout their attendance. Birth weight centile was calculated according to gestational age and weight at delivery^17^ using GROW software (http://www.gestation.net/cc/about.htm). At the time of serum sampling, patients with pruritus used a horizontal visual analogue score^18^ (0‐100 mm) to quantify in millimeters the worst itch symptoms experienced over the previous 24 hours. The marked point was measured, and the distance in millimeters from 0 (no itch) was converted to an itch score from 0 to 100. The itch analogue score quantified severity of pruritus but did not specify the physical location and extent of the itch.

Prior to analysis, participants were grouped according to retrospective assessment of their diagnosis of ICP at any point during the pregnancy.

#### Serum Bile Acid and Progesterone Sulfate Analysis by High‐Performance Liquid Chromatography‐Tandem Mass Spectrometry

Internal standards (100 ng of d4‐glycholic acid, d4‐glycochenodeoxycholic acid, d4‐glycodeoxycholic acid, d4‐glyco‐UDCA, d4‐glycolithocholic acid, d4‐UDCA, d4‐lithocholic acid (all from Qmx Laboratories, Essex, UK), d5‐cholic acid (Toronto Research Chemicals, Toronto, Canada), and d4‐taurocholic acid (TLC PharmaChem, Vaughan, Canada), dissolved in 40 μL methanol [MeOH]) were added to 100 μL of serum and vortexed. Acetonitrile (800 μL) was added to precipitate proteins. After vortexing and centrifugation, the supernatant was dried in a stream of nitrogen and then first taken up in 125 μL MeOH, followed by 125 μL of an aqueous solution containing 40% MeOH, 0.02% formic acid, and 10 mmol/L ammonium acetate. Before injection 75 μL of the sample was transferred to new vials and 80 μL of the following mix was added: three parts of MeOH and one part of an aqueous solution containing 40% MeOH, 0.02% formic acid, and 10 mmol/L ammonium acetate.

Ten microliters of this mixture was analyzed on a high‐performance liquid chromatography Alliance 2695 system coupled to a Xevo TQ mass spectrometer (Waters, Manchester, UK) using a SunFire C18 (4.6 × 100 mm, 3.5 μm) column (Waters) and gradient elution with 0.01% formic acid and 5 mmol/L ammonium acetate in water along with 0.01% formic acid + 5 mmol/L ammonium acetate in MeOH as the mobile phase. Cone voltage was 60 V and collision energy 18 eV for unconjugated bile acids, 60 V and 29‐43 eV for glycine conjugates, and 88 V and 56‐65 eV for taurine conjugates, respectively. Analytes were detected using selected ion monitoring and quantified by internal standard methods. The desolvation temperature was 650°C, and the source temperature was 150°C. Selected reaction monitoring was used with dwell times of 100 ms. Analytes were quantified using deuterized internal standards except for progesterone sulfates for which d4‐glyco‐UDCA was used. Results were calculated as response (area _analyte/_area_internal std_). Retention times and response curves of bile acids listed (Supporting Table S1) were evaluated from reference compounds obtained from Sigma; 5β‐pregnan‐3β‐ol,20‐one,3‐sulfate (pregnandiol‐3‐sulfate), 5α‐pregnan‐3α‐ol,20‐one,3‐sulfate (allopregnandiol‐3‐sulfate), and 5α‐pregnan‐3β‐ol,20‐one,3‐sulfate (epiallopregnandiol‐3‐sulfate) were obtained from Steraloids, USA; 5β‐pregnan‐3α,20α‐diol‐3‐sulfate, 5β‐pregnan‐3α,20α‐diol‐disulfate, and 5α‐pregnan‐3α,20α‐diol‐disulfate were from Sai Advantium, India. 5α‐Pregnan‐3β,20α‐diol‐disulfate was tentatively identified as the remaining isomer from its retention times and mass spectrum. 5β‐Pregnan‐3α,20α‐diol‐disulfate and 5α‐pregnan‐3α,20α‐diol‐disulfate coeluted at all of the conditions tested. Using this system, we observed less than 10% intra‐assay variability when rerunning the same sample. These assays were performed in the Department of Molecular and Clinical Medicine, University of Gothenburg, Gothenburg, Sweden.

#### Measurement of Serum Autotaxin Activity

Autotaxin activity was measured as described.^7^ Serum was incubated with 1 mmol/L lysophosphatidylcholine 14:0, 500 mmol/L NaCl, 5 mmol/L MgCl_2_, 100 mmol/L Tris (pH 9.0), and 0.05% Triton X‐100 for 60 minutes at 37^o^C. Liberated choline was detected using choline oxidase (2 U/mL), horseradish peroxidase (1.6 U/mL), and homovanillic acid, with emitted fluorescence recorded using a NOVOstar analyzer. Using this system, we observed less than 10% inter‐assay and intra‐assay variance. The autotaxin assay was performed at the Academic Medical Centre, Amsterdam, The Netherlands.

#### Cyclic Adenosine Monophosphate Bioluminescence Resonance Energy Transfer CAMYEL Assay

The bioluminescence resonance energy transfer CAMYEL (cAMP sensor using YFP‐Epac‐RLuc) cyclic adenosine monophosphate (cAMP) sensor permits quantification of intracellular cAMP concentrations with high sensitivity and a broad dynamic range^19^ and has been used previously to measure TGR5 signaling in cells.^20^ HEK293 cells that stably express the TGR5 receptor were generated using the FLP‐In system (Invitrogen). The characterization of these cells has been described.^21^ HEK293 and HEK‐HA‐TGR5 cells (4 × 10^6^ per 10‐cm plate) were transfected with 4 μg of complementary DNA (cDNA) encoding the CAMYEL sensor. Cells were transfected using polyethylenimine with a 6:1 polyethylenimine:cDNA ratio in 500 μL of 0.15 M NaCl. The polyethylenimine:cDNA mixture was added to the cells in the 10‐cm plate, and cells were incubated overnight in 5% CO_2_ at 37°C in Dulbecco's modified Eagle's medium supplemented with 10% fetal bovine serum. Cells were washed in phosphate‐buffered saline and incubated in 1 mL of versene for 10 minutes. Cells were suspended in Dulbecco's modified Eagle's medium and 10% fetal bovine serum, plated onto poly‐d‐lysine‐treated 96‐well plates, and incubated overnight in 5% CO_2_ at 37°C. To test cAMP production, cells were washed in prewarmed Hank's balanced salt solution and then incubated in 80 μL Hank's balanced salt solution for 30 minutes at 37°C. Coelenterazine (NanoLight Technology, Pinetop, AZ; 10 μL of 10 μmol/L in Hank's balanced salt solution) was added, and cells were incubated in the dark for 10 minutes at 37°C. Luminescence for RLuc8 (480 nm) and YFP (530 nm) was measured using a microplate reader (PHERAstar Omega; BMG Labtech, Mornington, Australia). A 2‐minute baseline was established before addition of the agonists. cAMP production was measured for 10 minutes following addition of the agonists, forskolin (10 μmol/L), or vehicle. Baseline and vehicle control values were subtracted, and the bioluminescence resonance energy transfer signal was normalized as a percentage of the forskolin response. This assay was performed at Monash University, Parkville, Australia.

#### Scratching Behavior

Scratching behavior was studied in mice (C57BL/6 (wild‐type [WT]), *Tgr5* knockout [KO], male and female, 6‐10 weeks) as described.^9^ The fur at the base of the neck was shaved, and mice were placed in individual cylinders on a glass shelf. Mice were acclimatized to the experimental room, restraint apparatus, and investigator for 2‐hour periods on 2 successive days before experiments. After acclimatization, 20 μL of 100 μmol/L 5β‐pregnan‐3α‐20α‐diol‐sulfate (PM3S) or vehicle (1% dimethyl sulfoxide) was injected intradermally at the nape of the neck (vehicle WT n = 4, PM3S WT n = 5, PM3S *Tgr5‐*KO n = 4). Hind limb scratching to the injection site was video‐recorded for 120 minutes. Two observers unaware of test agents or genotypes quantified scratching behavior. One scratch was defined as lifting the hind limb to the injection site and then placing the paw on the floor, regardless of the number of strokes. If counts differed by more than three scratches over a 30‐minute period, both observers reevaluated the record. Results are expressed as scratching events during 60 minutes of observation.

#### Transactivation Assays

Huh7 cells seeded into 96‐well plates were transfected with 10.4 ng plasmid circular DNA (pcDNA)‐retinoid X receptor, 10.4 ng pcDNA‐FXRα2/pcDNA3.1 together with 10.4 ng and 40 ng pGL3‐IBAP‐Luc and pcDNA3.1‐green fluorescent protein using Fugene 6 transfection reagent (Promega) at a 3:1 ratio. Twenty‐four hours later, cells were washed and treated with 0 or 50 μM compound ± 0.5 μM GW4064 (Sigma‐Aldrich). After 24 hours, green fluorescent protein activity was measured (internal control for normalization) followed by the addition of Steadylite plus (PerkinElmer) to determine luciferase activity, both of which were measured in a PheraStar FS (BMG) plate reader. Transfection experiments were performed three times, and the results are shown as mean values of triplicates and standard deviations.

#### Statistics

For group 1, log transformations of data were undertaken and results are presented as ratios of the geometric mean values between groups and over time. Results were corrected for multiple measures and multiple markers being analyzed. Interval regression was used for each assay.

Trend tests were performed by analyzing the random‐effects interval regression on the logged concentrations, with interactions between patient groups and linear effects of time.

For group 2, patient demographic group results and visual analogue itch scores were compared using the Mann‐Whitney U test, and serial serum concentrations of progesterone metabolites using unpaired Student *t* test (Prism 6; Graphpad Software Inc.). Progesterone metabolite concentrations were log‐transformed prior to analysis due to nonnormally distributed data. Longitudinal comparisons between disease groups of pruritus scores with biochemical markers were performed using Stata software (version 11; StataCorp, College Station, TX). Confounding based on multiple measures and gestational effects was accounted for, and subsequent linear and logistic regression analyses were performed.

For the HEK‐HA‐TGR5 cAMP assays and murine scratching assays, results are expressed as mean ± standard error of the mean. Data were compared statistically using Graphpad Prism 6 for multiple groups analysis of variance and Tukey‐Kramer *post hoc* test. *P* < 0.05 was considered significant.

## Results

#### Progesterone Sulfates Are Prognostic Indicators of ICP

To establish whether progesterone sulfates can predict women at risk of ICP in early pregnancy before symptom onset, a group of ICP cases and uncomplicated pregnancy controls was used to establish gestational profiles of three sulfated progesterone metabolites (group 1; Table 1). We obtained the following progesterone sulfate standards, which were previously implicated in ICP based on analysis of gas chromatograhic/mass spectrometric spectra^22^, ^23^ and all of which were synthesized *de novo*: 5α‐pregnan‐3α,‐20α‐diol‐3,20‐disulfate (PM2DiS), 5β‐pregnan‐3α,‐20α‐diol‐3,20‐disulfate (PM3DiS) (Supporting Fig. S1).

**Table 1:** Clinical and Demographic Characteristics of ICP, PG and Control Cases in Two Groups Used to Evaluate Sulfated Progesterone Metabolites as Biomarkers

Characteristic	Group 1			Group 2		
	ICP (n = 35)	Control (n = 29)	*P*	ICP (n = 41)	PG (n = 14)	*P*
Age (years, ± SD)	33.4 ± 4.6	30.8 ± 4.8	0.02	32.7 ± 4.3	35.6 ± 4.3	0.01
Ethnic group, number (%)						
White	22 (63)	27 (93)	0.02	25 (61)	11 (79)	NS
Black	4 (11)	0	NS	4 (10)	0	NS
Asian	8 (23)	1 (3)	NS	9 (22)	1 (7)	NS
Other	1 (3)	1 (3)	NS	3 (7)	2 (14)	NS
Previous pregnancies ≥24 weeks, number (%)						
0	0	26 (90)	<0.01	11 (27)	2 (14)	NS
1	20 (57)	1 (3)	<0.01	18 (44)	9 (64)	NS
≥2	15 (43)	1 (3)	<0.01	11 (27)	2 (14)	NS
Unknown	0	1 (3)	NS	1 (2)	1 (7)	NS
Gestational age at diagnosis (weeks ± SD)	29^+1^ ± 6^+3^	n/a		30^+0^ ± 7^+2^	n/a	
Severity of ICP, number (%)						
Total bile acids = 10‐39.9 μmol/L	13 (37)	n/a		16 (39)	n/a	
Total bile acids ≥40 μmol/L	22 (63)	n/a		25 (61)	n/a	
Mean serum ALT, IU/L ± SD	88.3 ± 131.2	n/a		145.3 ± 197.2	29.4 ± 30.6	
Onset of labor, number (%)						
Spontaneous	5 (14)	16 (55)	<0.01	7 (17)	4 (29)	NS
Induced	14 (40)	8 (28)	NS	24 (59)	3 (21)	0.02
Prelabor cesarean section	13 (37)	2 (7)	0.02	8 (20)	6 (43)	NS
Unknown	3 (9)	3 (10)	NS	2 (5)	1 (7)	NS
Gestational age at delivery (weeks ± SD)	37^+0^ ± 1^+4^	39^+6^ ± 1^+2^	<0.01	37^+0^ ± 1^+3^	38^+2^ ± 0^+2^	<0.01
Preterm delivery <37/40, number (%)	12 (34)	0	<0.01	14 (34)	2 (14)	NS
Birth weight, kg ± SD	3.1 ± 0.4	3.5 ± 0.4	<0.01	3.1 ± 0.4	3.1 ± 0.6	NS
Birth weight centile, number ± SD	67 ± 28	49 ± 32	0.01	72 ± 25	47 ± 35	0.01

Mean serum ALT values based on levels detected in the first sample obtained from each ICP case. *P* value shown where a comparison resulted in statistical significance. Values are given as means, unless otherwise stated.

Abbreviations: ALT, alanine transaminase; n/a, not applicable; NS, not significant.

**Figure 1:**
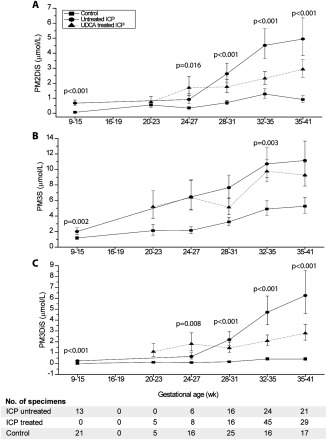
Gestational serum profiles of PM2DiS, PM3S, and PM3DiS in group 1. Panels A, B, and C show the mean concentrations of PM2DiS, PM3S, and PM3DiS, respectively, for serum samples obtained at different gestational time points from women with uncomplicated pregnancies (control, closed squares), untreated ICP (closed circles), and UDCA‐treated ICP (closed triangles). Error bars represent ± standard error of the mean. *P* values for gestational week category comparison of untreated ICP versus controls were determined by Student *t* test.

A comparison of geometric means across all gestational weeks for PM2DiS, PM3S, and PM3DiS revealed, respectively, 4.5‐fold, 2.0‐fold, and 12.2‐fold significant increases in serum concentrations in untreated ICP cases compared to pregnant controls (*P* < 0.001) (Fig. 1). Importantly, concentrations of PM2DiS, PM3S, and PM3DiS were supraphysiologically raised compared to normal pregnant controls by 10.6‐fold, 1.7‐fold, and 24.3‐fold, respectively, at weeks 9‐15 (*P* < 0.05), when 91% of these participants were asymptomatic, indicating their potential as predictive biomarkers for ICP. Concentrations of PM3S in ICP steadily increased at a constant rate from 9 to 41 weeks, whereas concentrations of PM3DiS and PM2DiS increased steeply from 24 to 41 weeks for the ICP group compared to controls (*P* < 0.05) (Fig. 1).

UDCA treatment improves maternal pruritus and biochemical derangements in ICP. UDCA significantly reduced PM2DiS and PM3DiS concentrations relative to untreated ICP women throughout the last trimester of pregnancy (*P* < 0.05). A trend analysis showed a significant change in the trend of PM3DiS with UDCA treatment in the third trimester of ICP compared to the untreated ICP group (*P* < 0.05), becoming similar to the pregnant control group trend (Fig. 1).

#### Progesterone Sulfate Concentrations Rapidly Resolve in ICP Serum Following Parturition

To establish whether progesterone sulfate concentrations persist following parturition in ICP, concentrations of progesterone metabolites in the last sample in ICP cases prior to parturition and postnatal samples collected thereafter were assayed in a subgroup of patients. Concentrations of PM2DiS, PM3S, and PM3DiS decreased rapidly following birth and normalized to almost undetectable levels as early as 12 days postpartum (Table 2).

**Table 2:** Maternal Concentrations of Progesterone Sulfates in the Last Serum Sample Prior to Parturition and in Subsequent Postnatal Serum Samples in ICP

Case	Gestational Day/Postnatal Day	PM3S (μmol/L)	PM3DiS (μmol/L)	PM2DiS (μmol/L)
1	GD 266	8.03	0.62	1.48
	PN+6	2.24	0.09	0.49
2	GD 241	2.75	1.28	1.27
	PN+66	0.00	0.00	0.00
3	GD 256	12.44	1.88	3.89
	PN+34	0.03	0.01	0.00
4	GD 255	7.22	2.19	3.30
	PN+12	0.04	0.03	0.09
5	GD 232	21.07	6.71	3.31
	PN+21	0.00	0.00	0.00
6	GD 269	2.28	0.52	1.25
	PN+40	0.00	0.00	0.00
7	GD 237	47.36	11.81	1.81
	PN+1	19.99	9.45	0.94
	PN+42	0.00	0.00	0.00
8	GD 261	9.85	4.76	5.94
	PN+40	0.02	0.00	0.00
9	GD 248	11.10	14.41	8.97
	PN+42	0.02	0.00	0.00
10	GD 252	20.04	5.31	4.90
	PN+13	3.74	3.44	1.72
11	GD 268	23.72	3.88	4.28
	PN+56	0.06	0.00	0.07
12	GD 255	15.64	5.60	6.04
	PN+1	9.29	5.15	6.00

Abbreviations: GD, gestational day; PN, postnatal.

#### Progesterone Sulfates Are Associated With Severity of Itch in ICP and Can Predict Its Subsequent Onset

To assess the involvement of progesterone sulfates in pruritus, we investigated the relationship between pruritus severity and serum concentrations of progesterone metabolites in women with pregnancy‐associated pruritus (Table 1). Serum PM2DiS, PM3S, and PM3DiS concentrations all differentiated women with ICP from PG (*P* < 0.05) (Table 3). Serum concentrations of PM3S (odds ratio [OR] = 6.1, 95% confidence interval [CI] 0.6‐11.5, *P* < 0.05) and autotaxin activity (OR = 1.4, 95% CI 0.3‐2.4, *P* < 0.05) were significantly associated with itch severity in ICP.

**Table 3:** Associations Between Biochemical Markers and Pruritus Scores and Their Ability to Differentiate ICP From PG

Biomarker	Association With Pruritus					
	ICP		PG		Ability to Identify ICP	
	Change in VAS (95% CI)	*P*	Change in VAS (95% CI)	*P*	OR (95% CI)	*P*
PM3S	6.1 (0.6‐11.5)	0.03	0.9 (−4.6–6.3)	NS	1.7 (1.1‐2.4)	0.01
PM3DiS	2.2 (−1.6–6)	NS	−2.5 (−6.2–1.2)	NS	2.1 (1.4‐3.4)	<0.01
PM2DiS	−0.3 (−5.3–4.6)	NS	−8.0 (−14.9–1.2)	0.03	1.7 (1.2‐2.5)	0.01
Autotaxin	1.4 (0.3‐2.4)	0.01	2.0 (−0.1–4.1)	NS	2.3 (2.1‐2.6)	<0.01

Linear regression results showing the effect of doubling biochemical markers and change in visual analogue score for ICP and PG and ORs for developing ICP. *P* value shown where a comparison resulted in statistical significance.

Abbreviations: NS, not significant; VAS, visual analogue score.

To determine whether progesterone sulfates could predict subsequent ICP, logistic regression was performed on PM2DiS, PM3S, and PM3DiS concentrations and autotaxin activity, using the first serum sample from women at presentation with pruritus and normal serum biochemistry (Table 4). PM2DiS and PM3DiS differentiated between the women who would subsequently develop ICP (*P* < 0.05, OR = 2.8, 95% CI 1.5‐5.2, and OR = 2.5, 95% CI 1.2‐5.4, respectively).

**Table 4:** Autotaxin, PM2DiS, and PM3DiS All Have the Ability to Predict ICP When Measured at the Time of Onset of Gestational Pruritus

ICP Marker	OR of Future ICP (95% CI)	*P*	Area Under ROC Curve
PM3S	1.70 (0.97‐3.01)	0.07	0.45 (0.23‐0.68)
PM3DiS	2.77 (1.48‐5.19)	<0.01	0.74 (0.55‐0.94)
PM2DiS	2.54 (1.18‐5.44)	0.02	0.72 (0.52‐0.92)
Autotaxin	2.22 (0.99‐4.86)	0.07	0.73 (0.52‐0.94)

Abbreviation: ROC, receiver operating characteristic.

**Figure 2:**
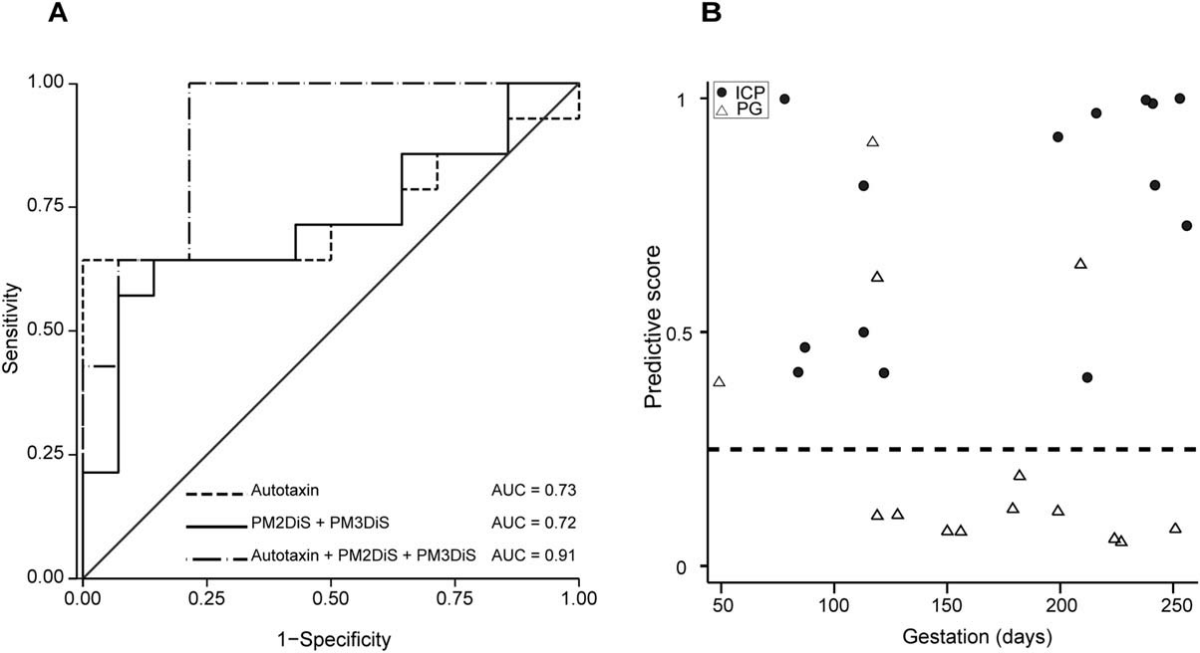
Progesterone sulfates and autotaxin can predict subsequent onset of ICP in pregnant women with pruritus. The receiver operating curves (A) improved toward an optimal area under the curve of 1.0 when biomarkers were evaluated in combination: PM2DiS + PM3DiS (complete line), autotaxin (dashed line), and PM2DiS + PM3DiS + autotaxin (dotted and dashed line). (B) A combined predictive score (PM2DiS + PM3DiS + autotaxin) of greater than 0.25 for individual samples plotted against the gestational day of sampling reliably predicted all ICP cases. Women who developed ICP (n = 14, closed circles) and PG (n = 14, open triangles) were reliably distinguished by this score; dashed line represents demarcation between the two groups. Abbreviation: AUC, area under the curve.

To refine a prediction algorithm, we evaluated whether a combination of markers could more reliably predict disease. PM2DiS, PM3DiS, and autotaxin in combination resulted in an improved area under the receiver operating characteristic (ROC) curve of 0.91 (95% CI 0.80‐1.00) in contrast to autotaxin (0.73, 95% CI 0.52‐0.94), PM2DiS (0.72, 95% CI 0.52‐0.92), or PM3DiS (0.74, 95% CI 0.55‐0.94) alone (Fig. 2A). Plotting this combination as a predictive score for the first serum sample from women presenting with PG enabled clear differentiation between those who would subsequently develop ICP and those who continue to have benign PG (Fig. 2B).

#### Progesterone Sulfates Are Supraphysiologically Raised in Early Gestation in Low‐Risk ICP Cases

We evaluated this predictive algorithm in a third group of asymptomatic pregnant women who gave serum samples at 11‐14 gestational weeks for a study of serum biomarkers to predict adverse pregnancy outcome (Table 5). Fifty‐four women from this group developed ICP in later pregnancy, and their progesterone sulfate concentrations were compared to those of 51 women with uncomplicated pregnancies. PM2DiS, PM3S, and PM3DiS concentrations were significantly raised in women with subsequent ICP (Table 5). Autotaxin did not predict ICP at this early gestation (area under the curve = 0.55, 95% CI 0.43‐0.66), while PM3DiS and PM2DiS in combination showed some predictive ability (area under the curve = 0.68, 95% CI 0.58‐0.78) (Supporting Fig. S2).

**Table 5:** Maternal Characteristics of Pregnancies Assessed in a Group of Low‐Risk Women Taken in the First Trimester of Pregnancy and Their Pregnancy Outcomes, With Levels of Serum Sulfated Progesterone Metabolites in These First‐Trimester Samples

Characteristic/Marker	Group 3		
	ICP (n = 54)	Control (n = 51)	*P*
Age, years ± SD	32 ± 5.4	31 ± 5.2	NS
Ethnic group, number (%)			
White	42 (78)	35 (69)	NS
Black	6 (11)	11 (22)	NS
Asian	5 (9)	3 (6)	NS
Other	1 (2)	2 (4)	NS
Previous pregnancies ≥24 weeks, number (%)			
0	28 (52)	26 (51)	NS
1	22 (41)	15 (29)	NS
≥2	4 (7)	10 (20)	NS
Onset of labor, number (%)			
Spontaneous	12 (22)	46 (90)	<0.01
Induced	34 (63)	3 (6)	<0.01
Prelabor cesarean section	8 (15)	2 (4)	NS
Gestational age at delivery, weeks ± SD	38^+3^ ± 1^+1^	40^+1^ ± 1^+1^	<0.01
Preterm delivery <37/40, number (%)	2 (4)	0	NS
Birth weight, kg ± SD	3.3 ± 0.5	3.4 ± 0.3	NS
Birth weight centile, number ± SD	60 ± 30	42 ± 23	<0.01
Stillbirth, number (%)	0	0	
Progesterone metabolite, μmol/L mean ± SEM			
PM3S	1.4 ± 0.1	1.1 ± 0.1	0.02
PM3DiS	0.3 ± 0	0.2 ± 0	<0.01
PM2DiS	2.8 ± 0.3	1.9 ± 0.2	<0.01
Total bile acids, μmol/L mean ± SEM	3.9 ± 0.4	4 ± 0.5	NS
Autotaxin activity, nmol/ml/min ± SEM	12.9 ± 1.2	11.6 ± 1.1	NS

*P* value shown where a comparison resulted in statistical significance. Values given as means, unless otherwise stated.

Abbreviations: NS, not significant; SD, standard deviation; SEM, standard error of mean.

#### Progesterone Sulfates Signal Through TGR5 to Mediate Itch

**Figure 3:**
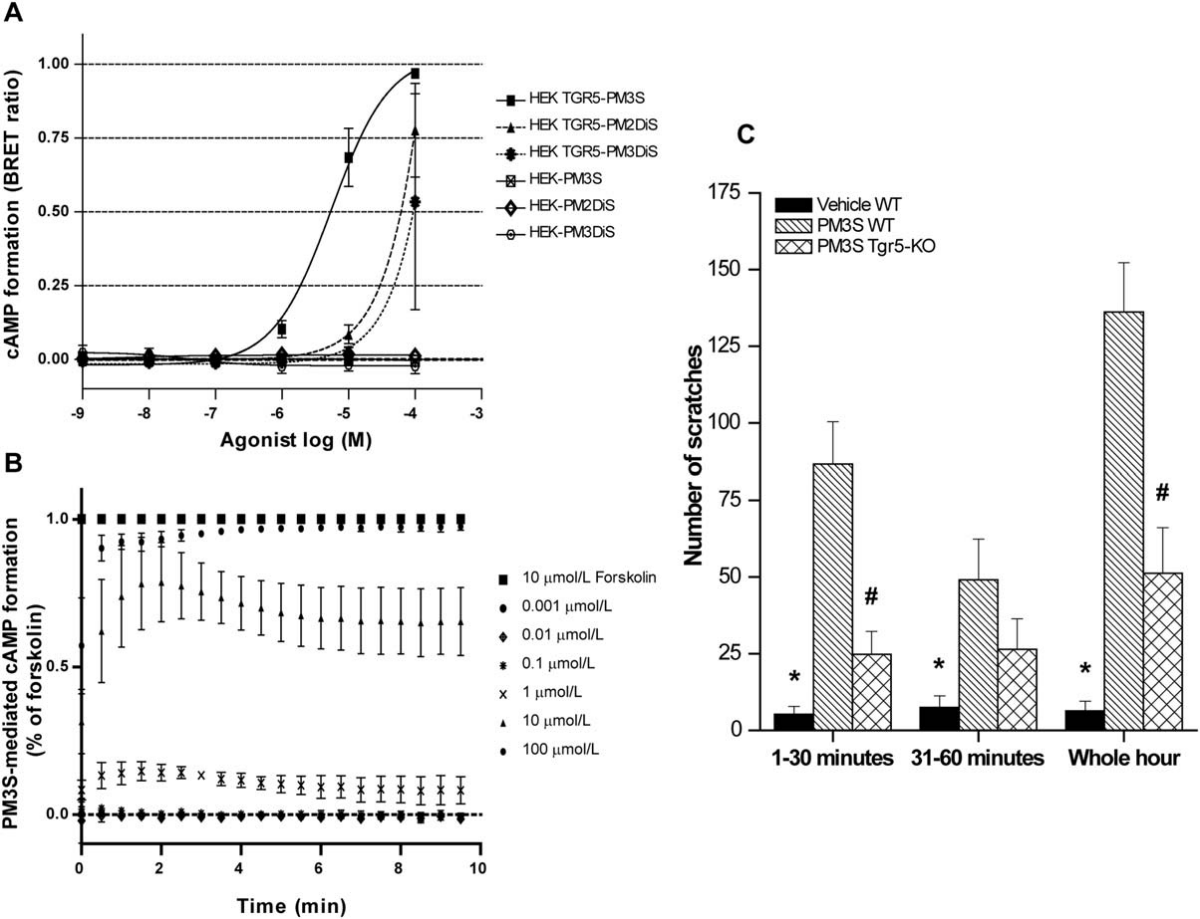
Progesterone metabolites can activate TGR5 and elicit a Tgr5‐mediated itch response in mice. cAMP formation was monitored over time in HEK293 cells expressing TGR5 or control cells that were treated with 10 μmol/L of forskolin or increasing concentrations of PM3S, PM3DiS, or PM2DiS. Data are presented as a dose response for all three compounds in both cell types (A) or time course for PM3S in TGR5‐expressing cells (B). Values represent mean ± standard deviation of n = 3. (C) Wild‐type or *Tgr5‐*KO mice were intradermally injected with vehicle or 20 μL of 100 μmol/L PM3S, and scratching events were counted for the indicated time periods. Values represent mean ± standard error of the mean of n ≥ 4. *P < 0.05 for vehicle versus PM3S‐administered mice scratch comparison; #*P* < 0.05 for PM3S WT versus PM3S *Tgr5*‐KO scratch comparison as determined by one‐way analysis of variance. Abbreviation: BRET, bioluminescence resonance energy transfer.

Activation of the G protein‐coupled receptor Tgr5 elicits an itch response in mice.^9^ We therefore hypothesized that progesterone metabolites associated with itch in ICP can activate TGR5 *in vitro*. HEK cells stably transfected with TGR5 or empty vector control cells were transfected with the CAMYEL cAMP sensor and treated with the cAMP inducer forskolin or increasing concentrations of PM2DiS, PM3S, and PM3DiS. In HEK‐TGR5 cells, PM3S elicited a cAMP response at concentrations of ≥1 μmol/L, whereas there was no cAMP response in control cells. PM3S stimulated a concentration‐dependent formation of cAMP with a 50% effective concentration of 5.5 μmol/L (Fig. 3A; and Supporting Fig. S3). Notably, these PM3S concentrations were demonstrated from 20 weeks' gestation in women with ICP (Fig. 1) and significantly associated with pruritus severity (Table 3). In contrast, PM2DiS and PM3DiS stimulated cAMP formation at extremely high concentrations. The temporal profile for the ≥1 μmol/L PM3S‐mediated cAMP response was consistent with the rapid actions of an activated G protein‐coupled receptor (Fig. 3B). We also excluded FXR as a possible mediator of the progesterone sulfate signal as all three progesterone sulfates were unable to either transactivate FXR or inhibit GW4064‐mediated FXR transactivity in an FXR‐reporter assay system (Supporting Fig. S4).

Because PM3S activates TGR5 in HEK‐TGR5 cells, we examined whether it could evoke Tgr5‐mediated scratching in mice. PM3S or vehicle (control) was intradermally injected into the nape of the neck of WT and *Tgr5‐*KO mice, and scratching behavior was measured for 60 minutes. In WT mice, PM3S stimulated a robust scratching response in the first 30 minutes, which was 16‐fold higher than that evoked by vehicle (*P* < 0.05) (Fig. 3C) and significantly blunted by three‐fold in *Tgr5*‐KO mice compared to WT mice (*P* < 0.05) (Fig. 3C). PM3S continued to stimulate scratching in WT mice from 30 to 60 minutes, whereas the response in *Tgr5*‐KO mice was attenuated after 30 minutes. Cumulatively over the whole hour, there was a 21‐fold increase in observed scratches in the PM3S‐challenged WT mice (*P* < 0.05), which was significantly abrogated in the *Tgr5‐*KO mice (*P* < 0.05).

## Discussion

Our results show that the sulfated progesterone metabolites PM2DiS, PM3S, and PM3DiS are prognostic for ICP as their concentrations are elevated during early gestation when patients are asymptomatic. Furthermore, UDCA treatment reduces the ICP‐associated elevation of disulfated progesterone metabolites. Interestingly, concentrations of progesterone sulfates decrease rapidly following birth, consistent with clinical reports of rapid resolution of pruritus in ICP.^24^ PM3S concentrations were associated with the pruritus of ICP, while all three progesterone sulfates were able to differentiate between women with pruritus in pregnancy secondary to ICP and those with benign PG. Combining PM2DiS, PM3DiS, and autotaxin activity enabled prediction of women who would subsequently develop ICP when they first started itching in pregnancy, prior to elevation in bile acids. Furthermore, concentrations of PM3S consistent with ICP were capable of mediating cAMP release in a TGR5‐dependent manner and resulted in a scratch response that was reduced in *Tgr5*‐KO mice.

This study has shown that PM3S is a likely pruritogen in ICP as concentrations consistent with ICP can activate TGR5 and mediate a Tgr5‐dependent itch. Although this result is based on a mouse model, Keitel et al. have also shown that progesterone sulfates can modulate the activity of TGR5 in other human tissues.^25^ We demonstrated that autotaxin and progesterone sulfates are associated with pruritus in ICP and PG, and it is likely that autotaxin‐mediated elevations in lysophosphatidic acid cause itch through a distinct mechanism from that of progesterone sulfate‐induced pruritus.

The demonstration that PM2DiS, PM3S, and PM3DiS are significantly raised in maternal serum prior to disease onset indicates that women with ICP are likely to have an underlying abnormality in phase 2 metabolism (conjugation) of progesterone or phase 3 (biliary excretion) of progesterone sulfates.^23^ As the progesterone sulfates that are supraphysiologically raised in ICP are agonists of the bile acid receptor TGR5, it is possible that they impact additional downstream gestational metabolic pathways mediated by this receptor.^26^ These results have the potential to provide insights into strategies to treat other cholestatic disorders complicated by itch, e.g., primary biliary sclerosis, primary sclerosing cholangitis, and drug‐induced liver injury. They are likely to also have a global impact as ICP is commoner in women of South Asian and South American origin.^5^, ^6^

At present there are no biomarkers for ICP in clinical use. The potential use of the predictive score to establish whether pregnant women with pruritus will develop ICP is enticing and should be evaluated in future prospective, well‐powered studies. This is important as the patient groups in the current study were all managed in a single specialist center and this may have introduced population bias. If the results are confirmed in different populations, a feasible extension to this study would be to assay concentrations of urinary progesterone sulfates (Glantz et al.^11^) to identify a predictive score that can be used in early pregnancy to establish whether a woman with pruritus will develop this high‐risk disease. This could have wider clinical application with the development of high‐throughput urinary assays for progesterone sulfates or similar laboratory tests for serum levels of progesterone sulfates and autotaxin. This will enable obstetricians to refer women for hospital care in a high‐risk setting or alternatively to reassure them that their pruritus is unlikely to have pathological consequences.

In conclusion, this study describes the mechanism of action of pruritogens that are prognostic for ICP, which has the potential to enable obstetricians to diagnose ICP, a common metabolic disorder of pregnancy, prior to onset of symptoms or biochemical derangements.

Author names in bold designate shared co‐first authorship.

## Supporting information

Additional Supporting Information may be found at onlinelibrary.wiley.com/doi/10.1002/hep.28265/suppinfo.

Supporting Information
