# Supplementary material for: Prognostic and mechanistic potential of progesterone sulfates in intrahepatic cholestasis of pregnancy and pruritus gravidarum
Source: Hepatology. 2015 Dec 28;63(4):1287–98. doi: 10.1002/hep.28265 (PMC4869673; doi:10.1002/hep.28265)
Supplement: Supplementary file 1 — Supporting Information [file HEP-63-1287-s001.pdf]

Supporting Table 1. Retention times for compounds assayed by HPLC-MS/MS.

Compound	Ions	RT KI	RT (LC-flow 0.6mL/min)
T-αMCA	514 > 79.9	2.56	2.56
T-βMCA	514 > 79.9	2.74	2.74
T-UDCA	498 > 79.9	3.99	3.80
**d4-G-UDCA**	452 > 73.9	4.19	4.03
G-UDCA	448 > 73.9	4.19	4.03
T-CA	514 > 79.9	5.77	5.39
**d4-GCA**	468 > 73.9	6.07	5.81
G-CA	464 > 73.9	6.07	5.81
PM5S	396.8 > 96.9	6.14	5.83
Iso-UDCA	391 > 391	6.32	6.44
**d4-UDCA**	395 > 395	6.82	6.92
UDCA	391 > 391	6.85	6.94
HCA	407 > 407	7.01	7.11
HDCA	391 > 391	7.8	7.87
T-CDCA	498 > 79.9	8.05	7.69
G-CDCA	448 > 73.9	8.47	8.16
**d5-CA**	412 > 412	8.51	8.56
CA	407 > 407	8.51	8.58
T-DCA	498 > 79.9	8.86	8.50
G-DCA	448 > 73.9	9.28	8.97
T-LCA	482 > 79.9	11.23	10.92
**T-OCA**	526 > 79.9	11.58	11.27
**d4-GLCA**	436 > 73.9	11.65	11.41
G-LCA	432 > 73.9	11.67	11.45
CDCA	391 > 391	11.71	11.81
**G-OCA**	476 > 73.9	11.92	11.74
DCA	391 > 391	12.12	12.21
**OCA**	419 > 419	14.15	14.28
**d4-LCA**	379 > 379	14.32	14.45
LCA	375 > 375	14.34	14.47
αMCA	407 > 407		5.14
βMCA	407 > 407		5.63

Supporting Figures

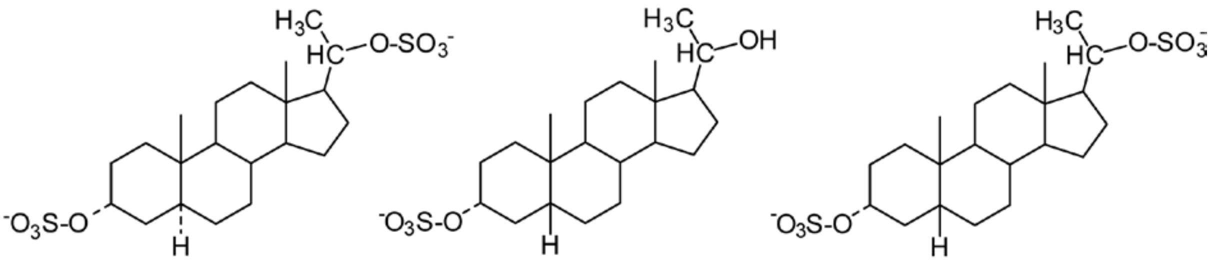

5α-pregnan-3α,-20α-diol-3,20-disulfate (PM2DiS) | 5β-pregnan-3α,-20α-diol-3-sulfate (PM3S) | 5β-pregnan-3α,-20α-diol-3,20-disulfate (PM3DiS)

Supporting Figure 1. Compound structures for the three synthesized progesterone metabolites.

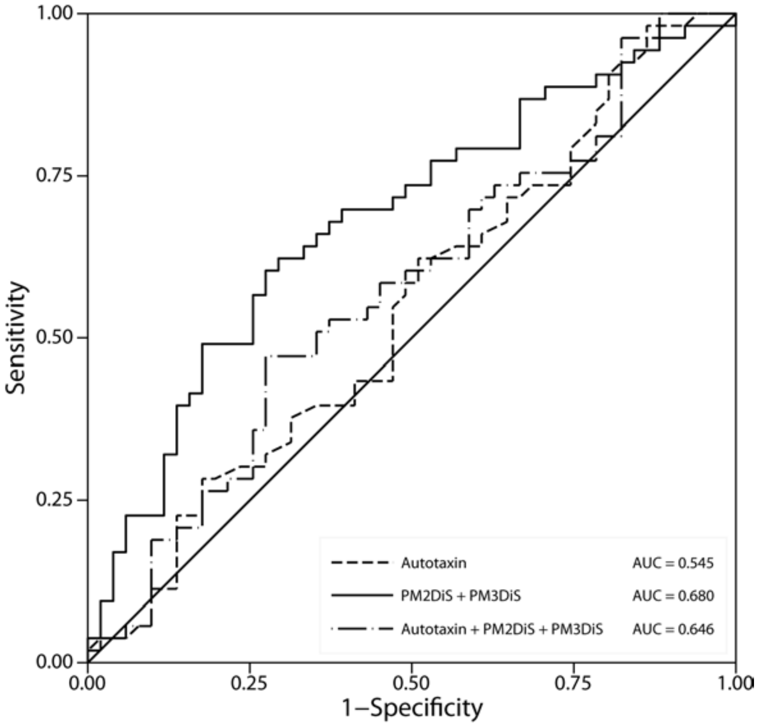

Supporting Figure 2. Disulfated progesterone metabolites can predict the onset of ICP using serum samples taken in the first trimester of pregnancy. Receiver operating curve for 11-14 week serum samples from women who subsequently developed ICP compared with control pregnancies (early prediction group). PM2DiS + PM3DiS (complete line), autotaxin (dashed line) and PM2DiS + PM3DiS + autotaxin (dotted and dashed line). AUC = area under curve.

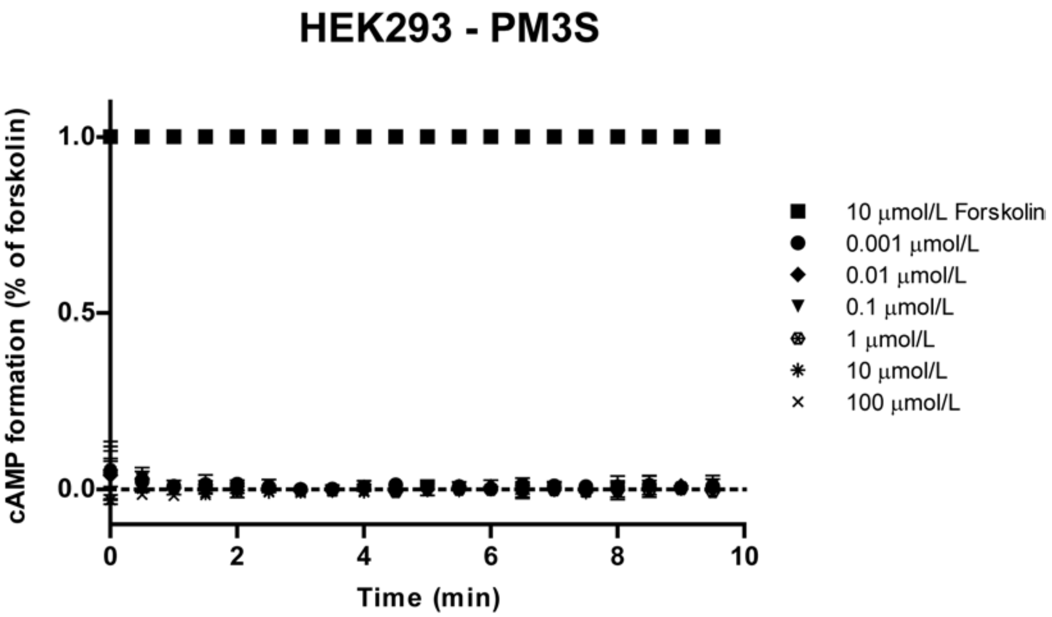

Supporting Figure 3. Wild type HEK293 lacking TGR5 cells transfected with a cAMP sensor do not respond to PM3S treatment. cAMP formation was monitored over time in HEK293 cells that were treated with a fixed 10 μmol/L dose of forskolin or increasing concentrations of PM3S. The data is presented as a time course. Values represent mean ± SD of n = 3.

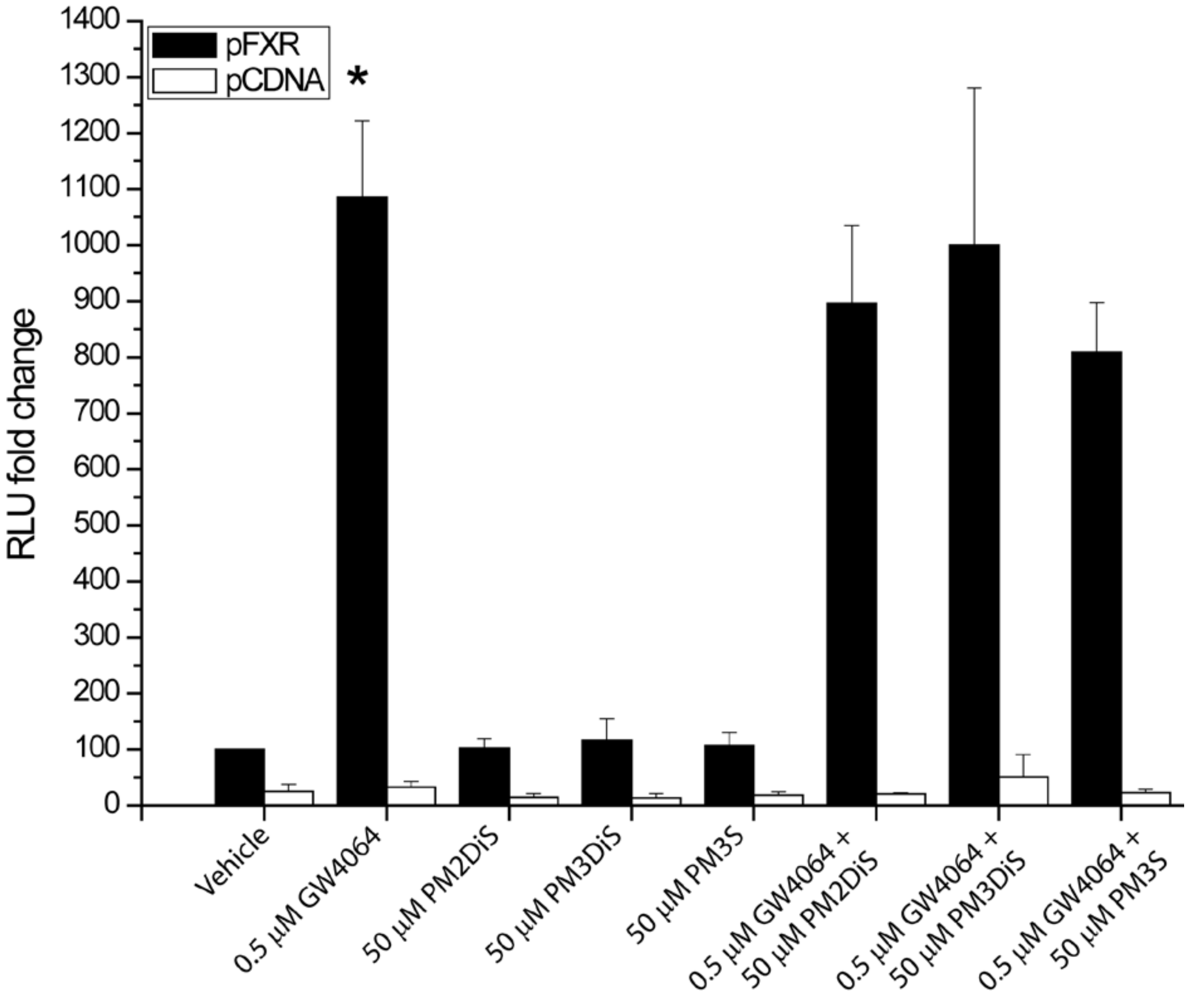

Supporting Figure 4. Progesterone sulfates do not modulate FXR activity. Huh7 cells transfected with pFXR or empty vector and the IBAP-luciferase reporter were treated with vehicle or 50 μM progesterone sulfate or 0.5 μM GW4064 ± 50 μM progesterone sulfates. *; $p<0.05$. Values represent mean ± SD of $n = 3$.
